# Quercetin Alleviates LPS-Induced Depression-Like Behavior in Rats *via* Regulating BDNF-Related Imbalance of Copine 6 and TREM1/2 in the Hippocampus and PFC

**DOI:** 10.3389/fphar.2019.01544

**Published:** 2020-01-17

**Authors:** Ke Fang, Hua-Rong Li, Xing-Xing Chen, Xin-Ran Gao, Ling-Ling Huang, An-Qi Du, Chuan Jiang, Hua Li, Jin-Fang Ge

**Affiliations:** ^1^ School of Pharmacy, Anhui Medical University, Hefei, China; ^2^ Anhui Province Key Laboratory of Major Autoimmune Diseases, Anhui Institute of Innovative Drugs, Hefei, China; ^3^ The Key Laboratory of Anti-inflammatory and Immune Medicine, Ministry of Education, Anhui Medical University, Hefei, China; ^4^ The First Clinical College, Anhui Medical University, Hefei, China

**Keywords:** quercetin, nesfatin-1, brain derived neurotrophic factor (BDNF), Copine 6, the triggering receptors expressed on myeloid cells (TREMs), synapsin-1

## Abstract

Quercetin is a polyphenol with multiple biological activities, and results of our preliminary study showed that it could shorten the immobility time of mice in the forced swimming test and tail suspending test. The aim of this study was to investigate its effects on the behavioral performance of lipopolysaccharide (LPS)-challenged rats and explore the potential mechanism. The results showed that intragastrical administration of quercetin (40 mg/kg) could improve the bodyweight gain of LPS-challenged rats, increase the saccharin preference index in the saccharin preference test and the novel arm preference index in the Y-maze, and decrease the immobility time in the FST. However, it showed no significant effect on the performance of LPS-challenged rats in the Morris water maze and the plasma concentrations of nesfatin-1, C-reactive protein (CRP), and IL-6. Results of western blot showed that the expression levels of BDNF, Copine 6, p-TrkB, and the triggering receptors expressed on myeloid cells (TREM) 1 were decreased in both the hippocampus and the prefrontal cortex (PFC) of LPS-challenged rats, while the expression of TREM2 was increased. The protein expression of synapsin-1 was decreased in the hippocampus without significant changes in the PFC. These imbalance protein expressions could be balanced by treatment with quercetin. The results suggested that quercetin could alleviate LPS-induced depression-like behaviors and impairment of learning and memory in rats, the mechanism of which might be involved with regulating the BDNF-related imbalance expression of Copine 6 and TREM1/2 in the hippocampus and the PFC.

## Introduction

Depression is a prevalent and recurrent mental abnormality that affects human health and economical development worldwide. It has been reported that during 2013–2016, 8.1% of the American adults aging 20 and above suffer from depression in a given 2-week period (Brody et al., 2018). Since first discovered in 1950s, most of the antidepressants are designed to correct the imbalances of neurotransmitters, which are taken as the key factor of regulating mind and emotion. Unfortunately, they are not effective for all the depression patients, and almost all the classical antidepressants share the same defect of long latency. Although the finding of the robust and rapid-onset antidepressant effects of ketamine has shifted the direction of antidepressants research and development (Dhir, 2017), and been taken as the single most important advance in the treatment of depression in over 50 years (Singh et al., 2017), it should be taken into account of the adverse effects including not only the cardiovascular symptoms, but also hallucinations, confusion, and irrational behaviors. Thus, it is urgent to further investigate the pathogenesis of depression and explore new effective antidepressants with less side effects and shorter acting period.

Inflammation has been long taken as one of the cornerstones in the development of depression (Jeon and Kim, 2017). Higher incidence of depression has been found in patients with infection, and depression patients present increased levels of pro-inflammatory cytokines and decreased level of brain-derived neurotrophic factor (BDNF) (Felger and Lotrich, 2013). C-reactive protein (CRP) and Interleukin 6 (IL-6) are common inflammatory markers, and elevated levels of CRP and IL-6 are associated with increased risk for psychological distress and depression (Wium-Andersen et al., 2013; Khandaker et al., 2014). Consistently, results of the animal studies reported that administration of lipopolysaccharide (LPS) could significantly elevate the IL-6 mRNA expression in both the brain and the spleen (Szot et al., 2017), resulting in depressive-like behavior and hippocampal microglial activation (Yamawaki et al., 2018). Moreover, the elevated plasma concentrations of CRP and IL-6 in patients with major depressive disorder are reported to be positively related to the increased plasma nesfatin-1 level (Xia et al., 2018), which is an anorexigenic molecule localized widely in the brain and peripheral tissues with multiple biological activities including regulation of feeding and mood (Weibert et al., 2019). Consistently, in our previous study (Ge et al., 2015a), administration with nesfatin-1 could induce depression-like behaviors, accompanied with the increased plasma concentrations of IL-6 and CRP. Moreover, the antidepressant-like effect of ibuprofen, which is one of the common used nonsteroidal anti-inflammatory drugs (NSAIDs), has demonstrated in animal studies (Mesripour et al., 2019; Seo et al., 2019). These findings support an overlapping pathobiology between inflammation and depression (Merali et al., 2003).

It is important to notice that the disruption of BDNF/TrkB signaling pathway and impairment of neuronal plasticity have been implicated in the pathogenesis of depressive disorders (Erickson et al., 2012). Hippocampus and prefrontal cortex (PFC) are key brain regions involved in the pathogenesis of depression and the antidepressant effects. Reduced serum BDNF levels, together with altered expression of BDNF and its high-affinity receptor tropomyosin receptor kinase B (TrkB) in the hippocampus and PFC, have been found in depression state and could be reversed by effective treatment with antidepressants (Gawali et al., 2016; Song et al., 2019; Seo et al., 2019). Results of our previous studies have suggested a positive relationship between the hippocampal expression of BDNF and Copine 6, synaptotagmin I, and synapsin I in stressed rats (Han et al., 2018). Copine 6 is another important molecular that plays a vital role in regulation of synaptic plasticity (Reinhard et al., 2016), *via* alternating the neurotransmission process (Liu et al., 2018) or morphology (Burk et al., 2018). Knockout of Copine 6 could induce a deficiency of hippocampal long time potentiation (LTP) and learning and memory in mice (Xu et al., 2015). However, little is known about their alternations in LPS-challenged rats.

The triggering receptors expressed on myeloid cells (TREMs) are a family of activating receptors, potentially may be manipulated to alter the inflammatory response (Bouchon et al., 2001). Inflammatory conditions that alter the balance in TREM expression could exert an important influence on homeostatic activity (Owens et al., 2017), resulting in different outcomes in different diseases (Konishi and Kiyama, 2018). Recent years, much attention has been paid to the role of TREM1/2 in the neuropsychiatric diseases including Alzheimer's disease (AD), although the results are not always consistent (Jiang et al., 2016; Suarez-Calvet et al., 2019; Jiang et al., 2017; Sayed et al., 2018). In our previous research, the protein expressions of TREM2 in the hippocampus are different between rats aging 2 months and 6 months, accompanied with a different performance in behavior tasks. These results suggest that the function of TREMs could be affected by a variety of factors, such as disease duration and stage (Jay et al., 2017).

Querctin is a polyphenol derived from many kinds of plants with multiple activities including antiinflammation and neuroprotective effects. Our previous results demonstrated that it could bind to beta amyloid (Aβ)1-40 and Aβ1-42 in monomer or fibril state. Recently, it has been reported that quercetin could protect against stress-induced anxiety- and depression-like behavior and improve memory in male mice (Samad et al., 2018). Consistently, results of our preliminary study showed that it could shorten the immobility time of mice in the forced swimming test (FST) and tail suspending test, though the mechanism remains unclear.

The aim of the present study is to investigate the effects of quercetin on LPS-induced depression-like behavior and explore its potential mechanism, especially its effect on the BDNF-related protein expression of the key molecules in the hippocampus and PFC.

## Materials and Methods

### Drugs

**Figure 1:**
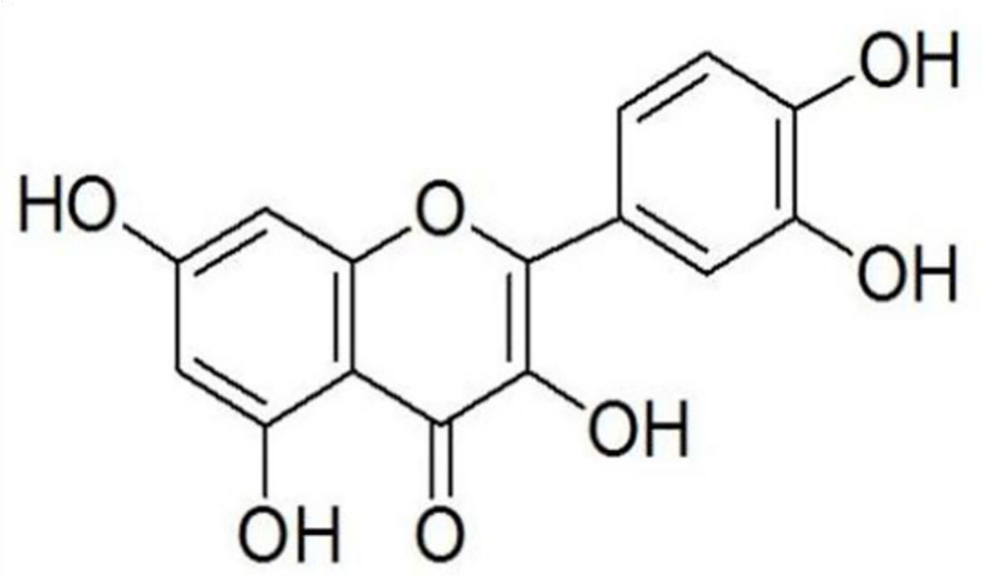
The structure of quercetin.

Quercetin was provided by Haoyang Biotechnology Co, LTD. (Xi'an, Shanxi, China) with the purity 95%, and the structure of quercetin is shown in **Figure 1**. Fluoxetine Hydrochloride (Prozac) was purchased from Eli Lilly Pharmaceuticals. Ibuprofen was the production of Renfu pharmaceutical co., LTD (Yichang, Hubei, China).

### Animals

**Figure 2:**
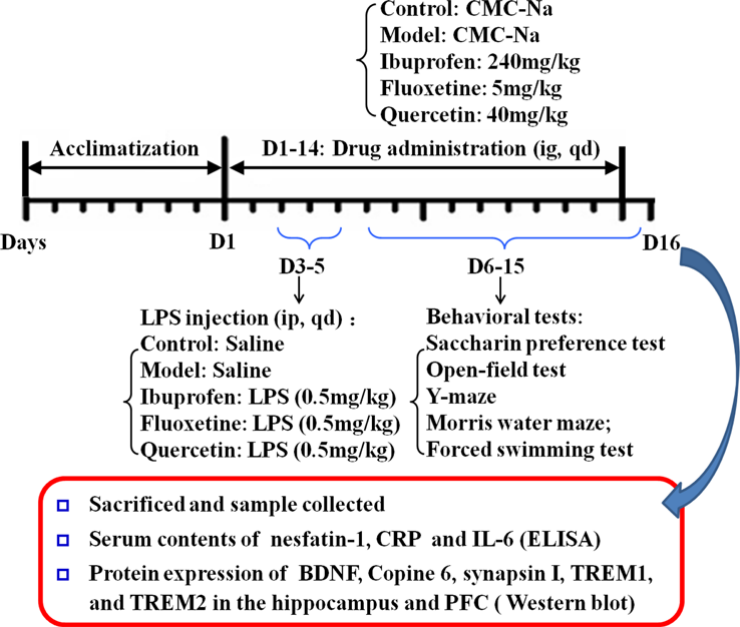
Schedule of the experimental design.

Fourty-five male Sprague-Dawley rats, aged 2 months, were purchased from Anhui Experimental Anhui Center of China. The rats were assigned randomly to five groups including an unhandled control group (Control), a LPS-injection group (Model), a ibuprofen treatment group (240 mg/kg), a fluoxetine treatment group (5 mg/kg), and a quercetin treatment group (40 mg/kg), with nine rats in each group. After a 7 days' adaptive breeding, rats were administered intragastrically the drugs daily with the dose mentioned above, while equal volume of 0.5% carboxymethyl cellulose (CMC) were given to rats in control group and model groups. During the 3^th^–5^th^ day, 0.5 mg/Kg LPS (from Escherichia coli, serotype 055:B5, Sigma-Aldrich) was administered through intraperitoneal injection to rats in model and drug-treated groups, and equivalent sterile normal saline solution was administered to rats in control group. The behavioral experiments were put into operation from the 5^th^ day. The schedule of the experimental design is shown in **Figure 2**.

Rats were raised and maintained under a 12-h light-dark cycle and housed 4–5 per cage with free access to food and water. The ambient temperature was maintained at 21°C–22°C with 50%–60% relative humidity. The experimental procedures were approved by the animal Care and Use committee at Anhui Medical University and complied with the National Institutes of Health Guide for the Care and Use of Laboratory Animals. (NIH publication No.85-23, revised 1985)

### Behavioral Tests

Behavioral tests were performed in a soundproof room with a neutral environment. All the rats in this study including the control ones received all the behavior tasks one by one. As the following description order, the first one was the saccharin preference test (SPT), followed by the open field test (OFT), then Y-maze and Morris water maze (MWM), and the last one was the FST. All the tests were carried out between 08:30 and 12:30, one hour after intragastric administration of solvent or drug, with matching between the groups. The observers were blind to the treatment. The behavioral tests were monitored and recorded by a digital camera interfaced to a computer running the ANY-maze video imaging software (Stoleting Co.,Wood Dale, IL, USA).

### Saccharin Preference Test

The SPT is one of the routine methods to evaluate the anhedonic behavior in rodents. After a 12-h period of food and water deprivation, all rats were housed solely and had free access to two bottles containing either plain water or 2% saccharin solution. After 6 h, the volumes of water and saccharin consumed were measured. The percentage of saccharin solution ingested, which is also named as saccharin preference index (SPI), is a measure to assess the sensitivity of rats to hedonia.

### Open Field Test

The OFT provides synchronous measures of locomotion, exploration, and anxiety. The apparatus of the test consisted of a black square arena with a length of 100 cm and a height of 30 cm. The floor was marked with white grids dividing it into 16 equally sized squares. During a 5-min observation period, rats were placed at one corner of the apparatus facing the wall. The total distance, duration in the center, and the frequencies of rearing and grooming were recorded. Rats were returned to their housed cages after test, and the open field was cleaned with 75% alcohol and permitted to dry between tests.

### Y-Maze

Y-maze consists of three identical arms with an angle of 120° between each other. Each arm was 10 cm × 48 cm × 20 cm (length× width× height). They were randomly designated as the start arm, the novel arm, and the familiar arm. The novel arm was blocked during the 1st trail but open during the 2nd trail, while the other two arms were always open. The maze was cleaned with 75% alcohol and permitted to dry between tests. The Y-maze test consisted of two trials separated by an intertrail interval to assess spatial recognition memory. The first trail allowed the rats 10 min to explore the start arm and the other arm. Twenty-four hours later, the second trail was conducted. The rat was placed back in the same starting arm, with free access to all the three arms for 5 min, and the percentage of the duration in the novel arm to that in both the novel and familiar arms was taken as the novel arm preference index.

### Morris Water Maze

MWM was used to test the spatial learning and memory. The maze was consisted of black circular pool (diameter 160 cm, height 50 cm, filled with water to 30 cm high containing black edible pigment at 21°C–22°C). A black circular platform (9 cm in diameter) was 2.0 cm below the water line in the center of one quadrant, and remained in the same position. Several constant and large visual cues surrounded the tank at a height of 60 cm to facilitate orientation.

The task consisted of a 3-day acquisition phase with four trails administered each day and a 1-day memory retention test phase. During the acquisition phase, the rat was placed in the water facing the wall at one random start location of four (north, south, east, and west, locating at equal distances from each other on the pool rim). Each rat was allowed to find the submerged platform within 60 s, and rest on it for 30s. If the rat failed to find the hidden platform within 60 s, it was guided to the platform and allowed to remain there for 30 s. The procedure was repeated for all the four start locations.

On the 4th day (the test phase), memory retention was determined in a single 60 s probe trial. The underwater platform was removed. The rats were placed into water from the opposite quadrant of the platform, facing the wall, and were permitted to explore the environment for 60 s *ad libitum*. Performance parameters of each rat including total swim distance, mean swim velocity, and the duration in each quadrant were monitored and recorded.

### Forced Swimming Test

The behavioral cylinder was 60 cm high with the diameter 25cm, filled with 30 cm of water and maintained at 24°C–25°C. The immobility time was recorded and rats were considered immobile when they did not make any active movements.

### Measurement of the Plasma Concentration of Nesfatin-1, IL-6, and CRP

Twenty-four hours after the last behavioral test, the rats were deeply anesthetized with chloral hydrate, and the blood was drawn from abdominal aorta. The plasma concentrations of nesfatin-1, IL-6, and CRP were measured using commercially available enzyme-linked immunosorbent assay (ELISA) kits (Nesfatin-1: Huamei Biotech. Co., LTD., Wuhan, China; IL-6 and CRP: Yuanye Biotech. Co., LTD., Shanghai, China) based on the manufactures’ instructions.

### Western Blot Assays

The hippocampus and the PFC of three rats from each group were promptly dissected, frozen in liquid nitrogen, and stored at −80°C. The tissue was homogenized in radio immuniprecipitation assay (RIPA) buffer (50mM Tris-HCl, pH 7.4, 0.1% SDS, 1% NP-40, 0.25% sodium deoxycholate, 150 mM NaCl, 1 mM EDTA, 1 mM EGTA, and 1 mM Na3Vo4). A protease inhibitor cocktail (Roche, IN, USA) and the phosphatase inhibitor PhosSTOP (Roche, IN, USA) were added before homogenization. The protein content was measured using a Lowry Protein Assay Kit (Meiji Biotech Co., Ltd., Shanghai, China). The same quantity (50 µɡ) of protein from each sample was loaded and separated by 15% SDS-PAGE and then transferred onto a polyvinylidene difluoride membrane (Amersham Biosciences, UK). The membrane was blocked with 5% skim milk for 1 h; incubated with antibodies targeting BDNF (1:1000; Abcam, Cambridge, UK), TrkB (1:10000; Abcam, Cambridge, UK), phosphorylated TrkB (p-TrkB) (1:10000; Abcam, Cambridge, UK), Copine 6 (1:1,000; Santa Cruz Biotechnology, Inc., Delaware, USA), synapsin-1 (1:1000; ImmunoWay, Newark, Delaware, USA), TREM-1 (1:1000; ImmunoWay, Newark, Delaware, USA), TREM-2 (1:1000; ImmunoWay, Newark, Delaware, USA), or β-actin (1:1000; Zhongshan Biotechnology, INC, Beijing, China) at 4°C overnight, and then incubated with a horseradish peroxidase-conjugated secondary antibody (1:2000) at 37°C for 2 h. The blots were developed with the Easy Enhanced Chemiluminescence Western Blot Kit (Pierce Biotechnology, Rockford, IL, USA). The protein bands were scanned and analyzed using Image J software (NIH). The TrkB/TrkB ratio was observed, and the expression of other proteins was normalized to β-actin and analyzed.

### Statistical Analysis

All statistical analyses were performed using SPSS (version 12.0.1, SPSS Inc., Chicago, IL, United States). Data are expressed as the means ± SEM and *P* < 0.05 was considered statistically significant. Between-group effects on the escaping latency in the MWM task were analyzed by repeated measures ANOVA with group and time as the factors. Statistical analyses of the effect of quercetin on other parameters were carried out using ANOVA followed by LSD posthoc tests. The correlation analysis was performed by Pearson's correlation test.

## Results

### Quercetin Administration Improved the Decreased Bodyweight Gain in LPS-Challenged Rats

**Figure 3:**
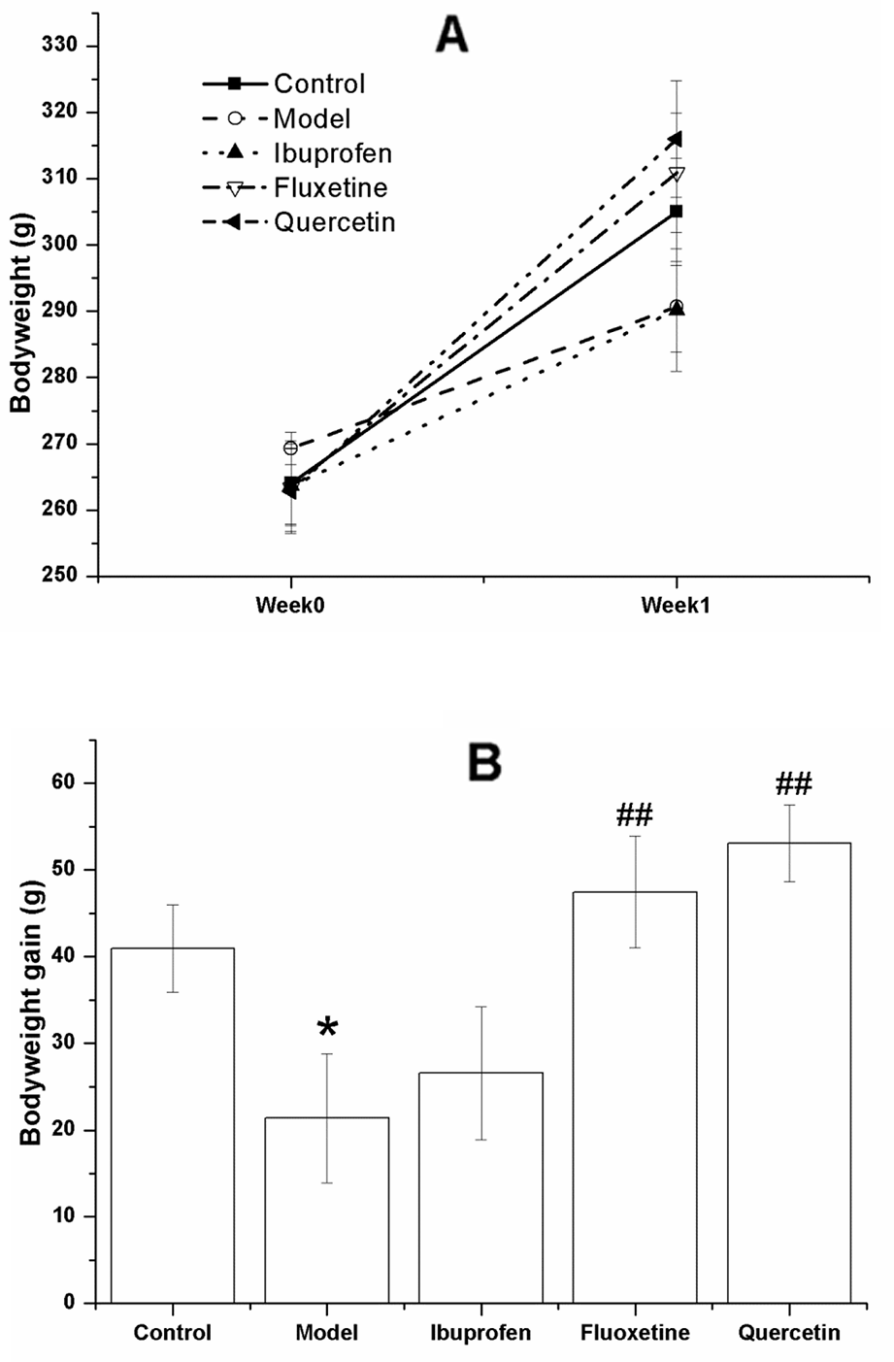
Effect of quercetin on the bodyweight and bodyweight gain in lipopolysaccharide (LPS)-challenged rats.The data are presented as the mean ± SEM, with nine rats in each group.There was no significant difference among groups with regard to the bodyweight **(A)**, but the bodyweight gain of the model rats in LPS-challenged group were less than that of the control ones, and this change could be reversed in the quercetin- and fluoxetine-treated group **(B)**. *P < 0.05, compared with the control group; ^##^ *P* < 0.01, compared with the model group.

**Figure 3** shows the bodyweight of the rats in different group before and after the test. As shown in **Figure 3A**, the bodyweight increased in all the groups, indicating that all the rats were growing. However, the bodyweight gain of the model ones were less than that of the control rats (F[4, 40] = 4.795, *P* = 0.031), and this change could be reversed by treatment with quercetin (F[4, 40] = 4.795, *P* = 0.005) or fluoxtine (F[4, 40] = 4.795, *P* = 0.001) (**Figure 3B**).

### Quercetin Administration Alleviated the Depression-Like Behavior in LPS-Challenged Rats

**Figure 4:**
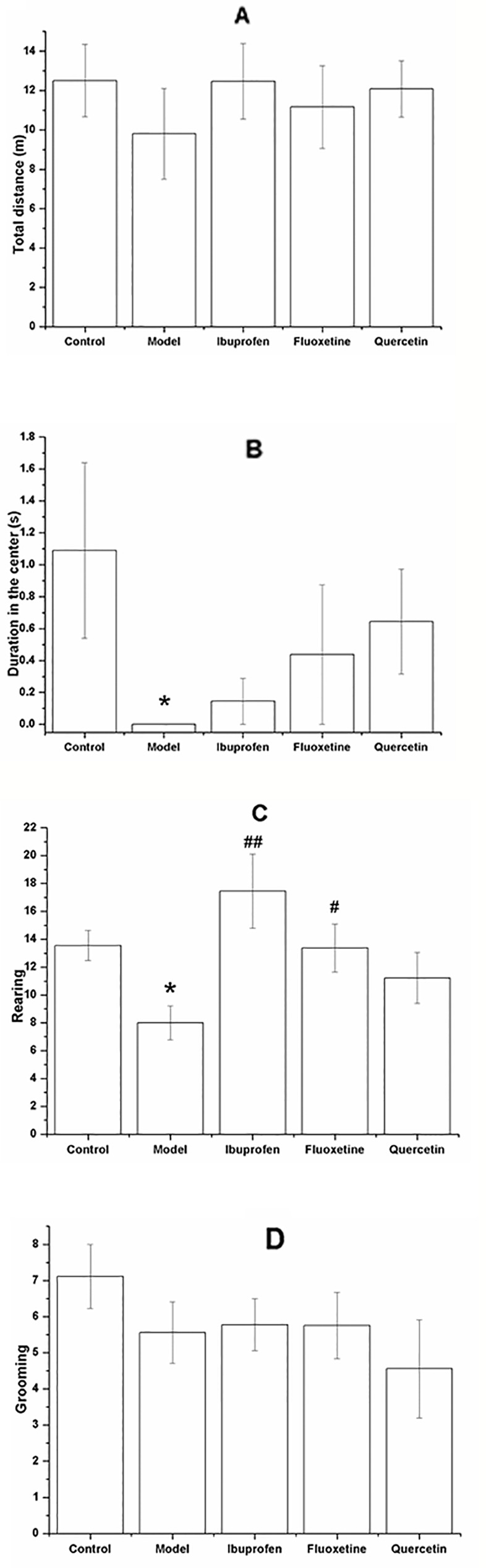
Effect of quercetin on the performance of lipopolysaccharide (LPS)-challenged rats in the open field test (OFT).The data are presented as the mean ± SEM, with nine rats in each group.In the OFT, there was no significant difference among groups with respect to the total moving distance **(A)** and grooming frequency **(D)**, but the duration in the center **(B)** and the rearing frequency **(C)** were significant decreased in the LPS-challenged group. Treatment with quercetin could increase the rearing frequency **(C)**.*P < 0.05, compared with the control group; ^#^ *P* < 0.05 and ^##^ *P* < 0.01, compared with the model group.

**Figure 5:**
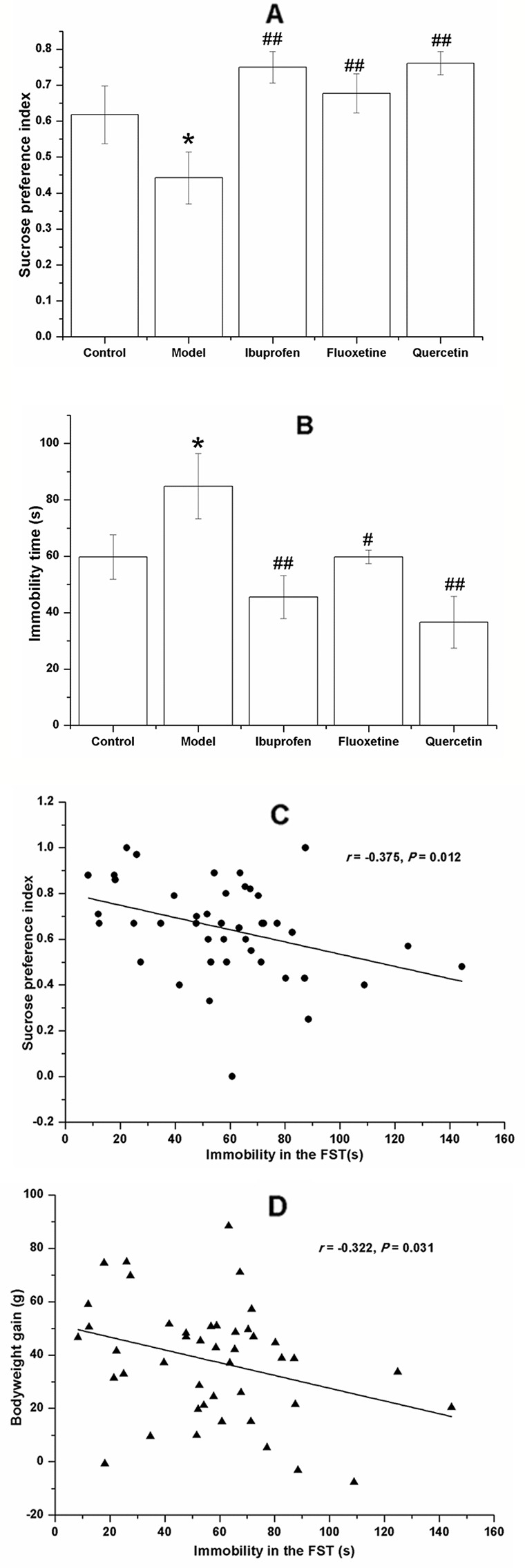
Effect of quercetin on the performance of lipopolysaccharide (LPS)-challenged rats in the saccharin preference test (SPT) and forced swimming test (FST). The data are presented as the mean ± SEM, with nine rats in each group. Compared with that in the control group, the sucrose preference index of rats in LPS-challenged group was lower **(A)** and the immobility time in the FST **(B)**. These changes could be reversed by treatment with quercetin, fluoxetine, and ibuprofen. The immobility time in the FST was negatively correlated to the sucrose preference index **(C)** and the bodyweight gain **(D)**. *P < 0.05, compared with the control group; ^#^ *P* < 0.05 and ^##^ *P* < 0.01, compared with the model group.

**Figure 4** shows the performance of rats in the OFT. Although there was no significant difference among groups with regards to the total distance (F[4, 40] = 0.377, *P* = 0.317; **Figure 4A**) and the grooming frequency (F[4, 40] = 0.918, *P* = 0.264; **Figure 4D**), the LPS-challenged rats spent less time in the center (F[4, 40] = 1.601, *P* = 0.030; **Figure 4B**) and showed less rearing frequency (F[4, 40] = 3.869, *P* = 0.031; **Figure 4C**), and treatment with ibuprofen (F[4, 40] = 3.869, *P* < 0.001; **Figure 4C**) or fluoxetine (F[4, 40] = 3.869, *P* = 0.042; **Figure 4C**) could significantly increase the rearing frequency. Compared with that in the control group, the model rats showed less SPI in the SPT (F[4, 40] = 4.809, *P* = 0.041; **Figure 5A**) and longer immobility time in the FST (F[4, 40] = 4.829, *P* = 0.039; **Figure 5B**). However, treatment with quercetin, fluoxetine, and ibuprofen could reverse the changes of SPI and immobility time (*P* < 0.05 or *P* < 0.01).

Results of the Pearson's correlation test showed that the immobility time in the FST was negatively correlated to the sucrose preference index (*r =* −0.375, *P =* 0.012; **Figure 5C**) and the bodyweight gain (*r =* −0.322, *P =* 0.031; **Figure 5D**).

### Quercetin Administration Improved the Performance of LPS-Challenged Rats in Y-Maze Without Significant Influence in the MWM

**Figure 6:**
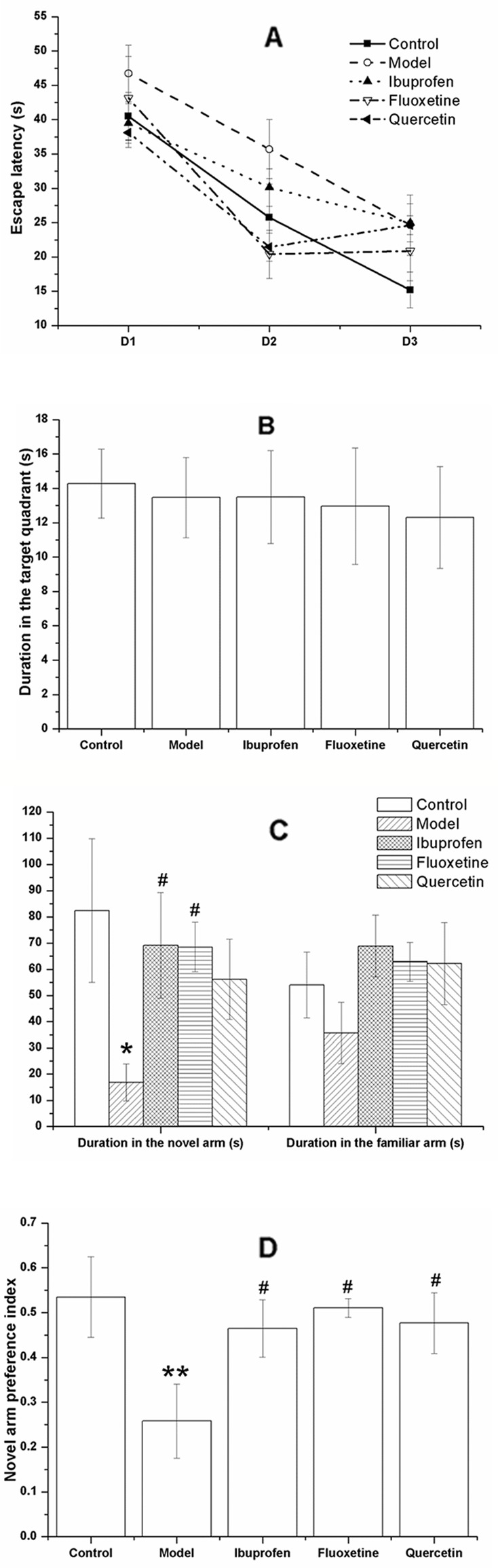
Effect of quercetin on the performance of lipopolysaccharide (LPS)-challenged rats in the Morris water maze (MWM) and Y-maze. The data are presented as the mean ± SEM, with nine rats in each group. The escape latency of all the rats was declined with each day during the acquisition phase **(A)**. Results of repeated measures showed that the training days, but not the experimental treatment had a significant effect on the escape latency. In the probe trail, there was no significant difference among groups of the duration in the target quadrant **(B)**. In the Y-maze, the duration or rats in the novel arm was decreased in the model group but significantly increase in the ibuprofen- and fluoxetine- treated group **(C)**. Treatment with quercetin could reverse the decreased novel arm preference index of LPS-challenged rats **(D)**. *P < 0.05 and **P < 0.001, compared with the control group; ^#^ *P* < 0.05, compared with the model group.

For all the rats studied in the experiment, the escape latency declined with each day during the acquisition phase, as is shown in **Figure 6A**. Analyzing the latency using repeated measures ANOVA with experimental treatment as the between-subject factor and training days as the within-subject factor, the results showed that only the training days (F[2, 39] = 65.426, *P* < 0.01), but not the experimental treatment (F[4, 40] = 2.077, *P* = 0. 102) had a significant effect on the escape latency, without a significant interaction between each other (F[8, 80] = 1.888, *P* = 0. 073). In the probe trail, there was no significant difference among groups with regard to the duration in the target quadrant (F[4, 40] = 0.136, *P* = 0.968; **Figure 6B**).

In the Y-maze task, the LPS-challenged rats spent less time in the novel arm than the controls (F[4, 40] = 2.099, *P* = 0.011; **Figure 6C**), together with a decreased novel arm preference (F[4, 40] = 2.669, *P* = 0.008; **Figure 6D**). These changes could be significantly reversed by treatment of fluoxetine or ibuprofen (*P* < 0.05). Treated with quercetin could increase the decreased novel arm preference index induced by LPS-challenge (F[4, 40] = 2.669, *P* = 0.028; **Figure 6D**).

### Quercetin Administration Did Not Change the Plasma Concentration of Nesfatin-1, CRP, and IL-6 in LPS-Challenged Rats

**Figure 7:**
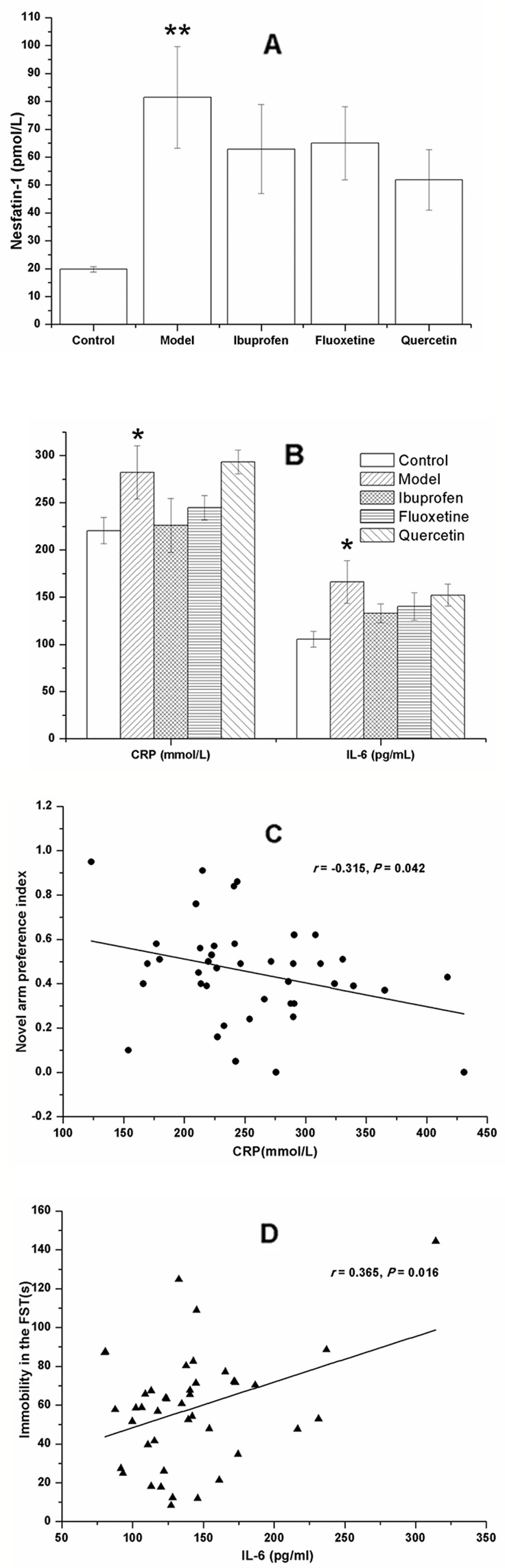
Effect of quercetin on the plasma concentrations of nesfatin-1, C-reactive protein (CRP), and IL-6 in lipopolysaccharide (LPS)-challenged rats. The data are presented as the mean ± SEM, with nine rats in each group. Compared with that of the control rats, the plasma concentrations of nesfatin-1, CRP, and IL-6 were all remarkably increased in the model group. There was no significant difference between the model group and the three groups with treatment of ibuprofen, fluoxetine, or quercetin **(A**, **B)**. Results of the Pearson's correlation test showed that the plasma CRP concentration was negatively correlated to the novel arm preference index **(C)**, and the plasma IL-6 concentration was positively correlated to the immobility time in the forced swimming test (FST) **(D)**. *P < 0.05 and **P < 0.001 compared with the control group.

As shown in **Figure 7**, the plasma concentrations of nesfatin-1 (F[4, 40] = 3.170, *P* = 0.003; **Figure 7A**), CRP (F[4, 40] = 2.610, *P* = 0.039; **Figure 7B**), and IL-6 (F[4, 40] = 2.442, *P* = 0.005; **Figure 7B**) were all remarkably increased in the model group, and there was no significant change after treatment with ibuprofen, fluxetine, or quercetin.

Results of the Pearson's correlation test showed that the plasma CRP concentration was negatively correlated to the novel arm preference index (*r =* −0.315, *P =* 0.042; **Figure 7C**), and the plasma IL-6 concentration was positively correlated to the immobility time in the FST (*r =* 0.365, *P =* 0.016; **Figure 7D**).

### Quercetin Administration Balanced the Protein Expression of BDNF, P-TrkB/TrkB, Copine 6, Synapsin-1, TREM1, and TREM2 in the Hippocampus and PFC of LPS-Challenged Rats

**Figure 8:**
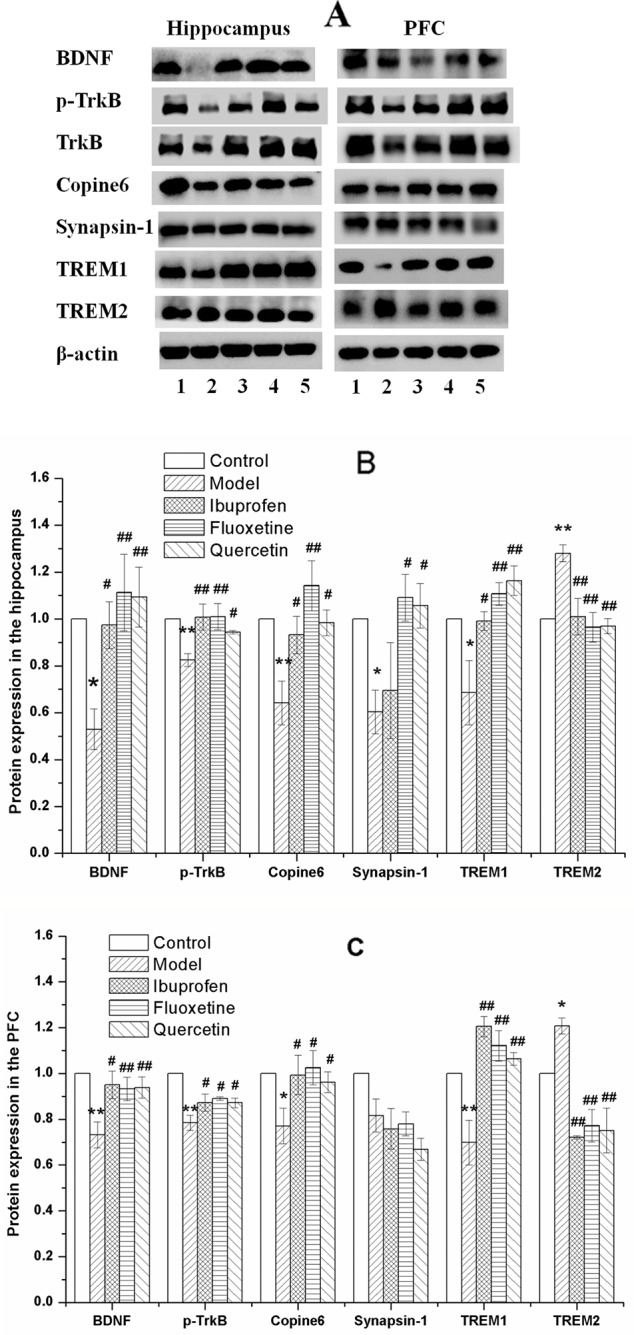
Effect of quercetin on the protein expression of brain derived neurotrophic factor (BDNF), Copine 6, TREM1, TREM2, and Synapsin-1 in the hippocampus and prefrontal cortex (PFC) of lipopolysaccharide (LPS)-challenged rats **(A)** shows a typical graph, and **(B)** and **(C)** show the statistical analysis of western blotting results. The data in **(B)** and **(C)** are presented as the mean ± SEM, with n = 3 for each group. The protein expression of BDNF, Copine 6, and TREM1 were decreased in both the hippocampus and the PFC of LPS-challenged rats, while the expression of TREM2 were increased, and the protein expression of synapsin-1 were decreased in the hippocampus without significant changes in the PFC. These changes could be balanced by treatment of ibuprofen, fluoxetine, and quercetin. *P < 0.05 and **P < 0.001, compared with the control group; ^#^ *P* < 0.05 and ^##^ *P* < 0.001, compared with the NAFLD model group. 1 control; 2 Model; 3 Ibuprofen; 4 Fluoxetine; 5 Quercetin.

**Figure 8** shows the key protein expression in the hippocampus and the PFC of rats in all the groups. As compared with that in the control group, the protein expression of BDNF, p-TrkB/TrkB, Copine 6, and TREM1 were decreased in both the hippocampus and the PFC of LPS-challenged rats, while the expression of TREM2 were increased, and the protein expression of synapsin-1 were decreased in the hippocampus without significant changes in the PFC. These changes could be balanced by treatment of quercetin.

**Figure 9:**
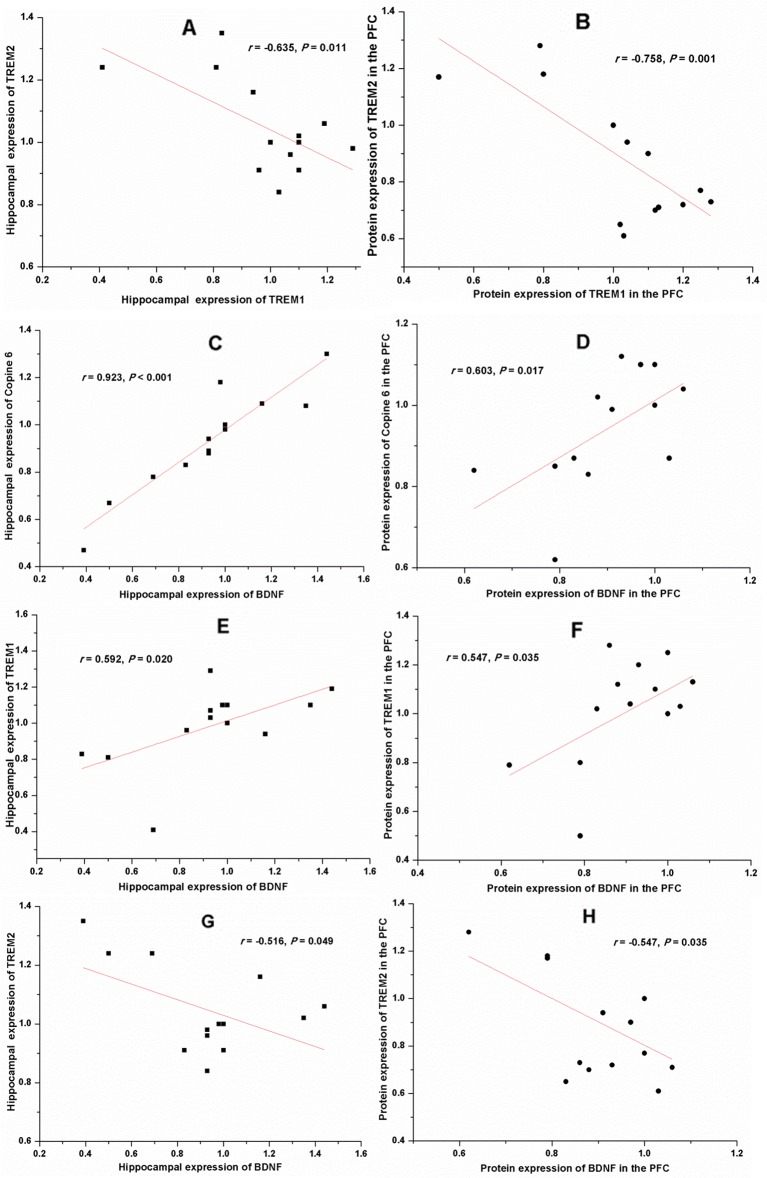
Correlation analysis of the protein expression of brain derived neurotrophic factor (BDNF), Copine 6, TREM1, and TREM2 in the hippocampus and the prefrontal cortex (PFC). A negative correlation was found between the protein expression of TREM1 and TREM2 in both the hippocampus **(A)** and the PFC **(B)**. The protein expression of BDNF was positively correlated to that of Copine 6 **(C**, **D)** and TREM1 **(E**, **F),** while negatively correlated to that of TREM2 **(G**, **H)**.

Apart from the negative correlation between the protein expression of TREM1 and TREM2 (Hippocampus: *r =* −0.635, *P =* 0.011, **Figure 9A**; PFC: *r =* −0.758, *P =* 0.001, **Figure 9B**), results of the Pearson's correlation test showed that the protein expression of BDNF was positively correlated to that of Copine 6 (Hippocampus: *r =* 0.923, *P* < 0.001, **Figure 9C**; PFC: *r =* 0.603, *P =* 0.017, **Figure 9D**) and TREM1 (Hippocampus: *r =* 0.592, *P =* 0.020, **Figure 9E**; PFC: *r =* 0.547, *P =* 0.035, **Figure 9F**), while negatively correlated to that of TREM2 (Hippocampus: *r =* −0.516, *P =* 0.049, **Figure 9G**; PFC: *r =* −0.547, *P =* 0.035, **Figure 9H**). A positive correlation was found between the expression of BDNF to that of synapsin-1 in the hippocampus (*r =* 0.613, *P =* 0.015), but not in the PFC (*r =* −0.060, *P =* 0.832).

## Discussion

In the present study, our results showed that quercetin could alleviate the depression-like behaviors induced by intraperitoneal injection of LPS in rats, as indicated by the increased SPI in the SPT and decreased immobility time in the FST. Moreover, the mechanism of its antidepressant-like effects might be associated with its ability in regulating the BDNF-related imbalance of Copine 6 and other synaptic plasticity-related protein expression in the hippocampus and PFC. Additionally, although administration of quercetin could not improve the performance of LPS-challenged rats in the MWM task, it increased the novel arm preference index in the Y-maze.

Inflammation is an important pathogenic factor involved with the development of depression. LPS is a common agent used as inflammatory stimuli. In spite of the different experimental protocol with different dose, frequency or administrating route, it has been reported that LPS-challenged rodents present depression-like behavior (Szot et al., 2017) (Wickens et al., 2017; Yamawaki et al., 2018) with a sex-specific Effect (Millett et al., 2019). In line with these findings, in our present study, LPS-challenged rats showed less bodyweight gain, less exploratory behavior, anhedonia, and despair behaviors. Using directional heading error as the evaluation index, Kupferschmid and his colleagues reported a selective, age-dependent spatial learning impairment in LPS-administered rats (Kupferschmid and Therrien, 2017). In the present study, although there was no significant difference among groups in the MWM tasks, the LPS-challenged rats showed a decline of novel arm preference index in the Y-maze. Additionally, results of the Pearson's correlation test showed a close relationship among the different behavioral indicators. These results indicated again that LPS could be widely used to investigate the relationship between inflammation challenge and the impairment of neuropsychiatric behaviors including anxiety, depression, and memory deficit. As a common used NASID, the antidepressant effect of ibuprofen has been demonstrated in several animal models and human studies (Saleh et al., 2014; Kohler et al., 2015; Mesripour et al., 2019; Seo et al., 2019). Consistently, our results showed that treatment with ibuprofen could increase the rearing frequency and SPI of LPS-challenged rats, together with the decrease of immobility time in the FST. The results suggested again the close relationship between inflammation and depression.

As an established antidepressant, fluoxetine was selected as a positive control in the present study. Consistent with its clinical application, fluoxetine has also been demonstrated to prevent LPS-induced increase in the immobility time in the FST and tail suspension tests (Taniguti et al., 2019). Unsurprisingly, in the present study, rats in fluoxetine-administered group showed improvement in both bodyweight gain and depression-like behaviors. Similar with the results of fluoxetine, administration of quercetin could also improve the bodyweight gain, increase the SPI in the SPT, and decrease the immobility time in the FST, although it had no effect on the performance in the OFT. Together with the findings that quercetin could reduce the immobility time of mice in the FST and tail suspend test in our preliminary study (attached in the **Supplementary Materials**) and relieve the depression-like behavior of stressed mice (Samad et al., 2018), our results suggested again the antidepressant effect of quercetin. Moreover, our results showed a task-related improvement effect of quercetin on the learning and memory.

Raised inflammatory markers are reported in depressed patients and taken as an important mediating factor for behavior, neural plasticity and brain structure (Valkanova et al., 2013; Lotrich, 2015). There is increasing evidence that inflammatory cytokines can induce or worsen depressive symptoms (Horowitz and Zunszain, 2015). Consistently, it has been reported in animal studies that LPS challenge could induce an increase of pro-inflammatory cytokine response (Ge et al., 2015b; Szot et al., 2017). In line with these findings, our results proved that plasma concentrations of CRP and IL-6 were remarkably increased in LPS-challenged rats. Moreover, our results showed a close relationship between the plasma inflammatory indicators and the behavioral performance. Furthermore, in line with the report that LPS could induce an elevation of plasma nesfatin-1 concentration in rats (Stengel et al., 2011), the plasma concentration of nesfatin-1 of LPS-challenged rats was also increased. These results suggested again the relationship between inflammation and depression. However, administration of quercetin could not reverse the increased plasma concentrations of nesfatin-1, CRP, and IL-6. Although we did not detect their alternation in the brain, our results indicated the hypothesis that there would be other mechanisms underlying the antidepressant-like effect of quercetin.

BDNF is well recognized for its neuroprotective functions, *via* binding and activating its high affinity receptor TrkB. The alternation of BDNF/TrkB signaling pathway has been demonstrated to play a key role in the pathophysiology of depression and in the therapeutic mechanisms of antidepressant (Zhang et al., 2016). In the present study, the protein expressions of BDNF, p-TrkB/TrkB, Copine 6, and synapsin-1were all reduced in the hippocampus and PFC of LPS-challenged rats, and a positive relationship was found between the protein expression of BDNF and Copine 6 or synapsin-1. Taking together with their important role in linking neuropsychiatric behaviors to synaptic plasticity (Jangra et al., 2014; Reinhard et al., 2016), these results indicated that BDNF-related imbalance expression of Copine-6 and synapse plasticity-associated proteins play a vital role in the depression-like behavior induced by LPS challenge. However, treatment with fluoxetine, ibuprofen, or quercetin could reverse these alternations. Therefore, it is plausible to infer that, apart from the reported mechanisms including modulating cytokines production and inhibiting oxidative stress (Sah et al., 2011), the antidepressant-like effect of quercetin might be associated with its ability in regulating the BDNF-related imbalance of key proteins expression involved in neuroinflammation and neuroplasticity.

The TREM family proteins are cell surface receptors with important roles in regulation of myeloid cell inflammatory activity, and different TREM receptors appear to have contrasting roles in controlling myeloid immune activity (Owens et al., 2017). Therefore the relative and coordinated regulation of their expression is important to understand, though the inconsistent reports made it more complicated. Although TREM-1 are demonstrated to amplify inflammation in sepsis (Bouchon et al., 2001), soluble TREM-1 has been taken as an antiinflammatory mediator in sepsis (Giamarellos-Bourboulis et al., 2006; Gibot and Massin, 2006). It has been reported that TREM2 can promote microglial cell survival by activating the Wnt/β-catenin signaling pathway (Zheng et al., 2017), and accelerate the removal of Aβ to reduce oxidative stress damage in hippocampal neurons (Fan et al., 2019). Upregulating the expression of TREM-2 could inhibit the apoptosis of hippocampal neurons (Liu et al., 2019), ameliorate neuropathology, and rescue spatial cognitive impairment (Jiang et al., 2014). Loss of TREM2 function increases amyloid seeding but reduces plaque-associated ApoE (Parhizkar et al., 2019), and aggravates spatial learning impairment in P301S transgenic mice (Jiang et al., 2015). However, it has also been reported that the ameliorative effect of TREM2 overexpression in microglia on the behaviors and neuropathological injuries including Aβ aggregation, neuroinflammation, and loss of neurons and synapses (Jiang et al., 2014). Similarly, Inconsistent or contrary effects of TREM2 on the microglial injury response and tau pathology were also reported (Bemiller et al., 2017) (Leyns et al., 2017), and even the effect would be varied by partial or complete loss of TREM2 function (Sayed et al., 2018). Additionally, different expression of TREM2 has also been found in the hippocampus between rats aging 2 months and 6 months in our previous study. In the present study, the LPS-challenged rats presented not only depression-like behaviors and impairment of learning and memory, but also the decreased expression of TREM1 and increased expression of TREM2 in the hippocampus and PFC. Despite the small sample size, our results suggested a negative relationship between the protein expression of TREM1 and that of TREM2. Moreover, these alternated expressions of TREM1 and TREM2 could be reversed by treatment of quercetin. These results suggested again the important role of TREMs in regulating the neurological behaviors and the potential effect of quercetin, although more details should be investigated thoroughly in the future.

There are several limitations in our study. Firstly, the multiple behavioral tasks were carried out in the same cohort of rats. Although the confounding factors including time, place, order have been taken into account and controlled, we cannot exclude absolutely the interactions among the behavioral tasks. Secondly, due to the limited data of continuous variables, the Pearson correlation test were carried out based on all the data together. Although it could increase the sample size and enlarge the variation of the observation indicators, making it more convenient and reliable to observe the relationship between the changes of the indicators. However, we cannot exclude the limitation by the influence of different treatments. More detailed investigation should be taken in our further study.

Taken together, our study revealed that quercetin could alleviate LPS-induced depression like behaviors and impairment of learning and memory in rats, the mechanism of which might be partly associated with its ability in regulating the BDNF-related imbalance expression of Copine 6 and TREM1/2 in the hippocampus and PFC.

## Data Availability Statement

The datasets generated for this study are available on request to the corresponding author.

## Ethics Statement

The experimental procedures were approved by the animal Care and Use committee at Anhui Medical University and complied with the National Institutes of Health Guide for the Care and Use of Laboratory Animals (NIH publication No. 85-23, revised 1985).

## Author Contributions

J-FG designed the study and wrote the protocol. H-RL wrote the first draft of the manuscript. KF and X-XC managed the literature searches and the statistical analyses. KF, L-LH, CJ, A-QD, and HL performed animal model experiments. X-XC and X-RG performed the gene expression experiments and wrote parts of the manuscript. All authors contributed to and have approved the final manuscript.

## Conflict of Interest

The authors declare that the research was conducted in the absence of any commercial or financial relationships that could be construed as a potential conflict of interest.
