# Corrigendum: Quercetin Alleviates LPS-Induced Depression-Like Behavior in Rats *via* Regulating BDNF-Related Imbalance of Copine 6 and TREM1/2 in the Hippocampus and PFC

**DOI:** 10.3389/fphar.2020.00518

**Published:** 2020-04-15

**Authors:** Ke Fang, Hua-Rong Li, Xing-Xing Chen, Xin-Ran Gao, Ling-Ling Huang, An-Qi Du, Chuan Jiang, Hua Li, Jin-Fang Ge

**Affiliations:** ^1^ School of Pharmacy, Anhui Medical University, Hefei, China; ^2^ Anhui Province Key Laboratory of Major Autoimmune Diseases, Anhui Institute of Innovative Drugs, Hefei, China; ^3^ The Key Laboratory of Anti-inflammatory and Immune Medicine, Ministry of Education, Anhui Medical University, Hefei, China; ^4^ The First Clinical College, Anhui Medical University, Hefei, China

**Keywords:** quercetin, nesfatin-1, brain derived neurotrophic factor (BDNF), Copine 6, the triggering receptors expressed on myeloid cells (TREMs), synapsin-1

An author name was incorrectly spelled as “Fang Ke.” The correct spelling is “Ke Fang”.

The authors apologize for this error and state that this does not change the scientific conclusions of the article in any way. The original article has been updated.

