# Supplementary figures and images for: Quercetin Alleviates LPS-Induced Depression-Like Behavior in Rats via Regulating BDNF-Related Imbalance of Copine 6 and TREM1/2 in the Hippocampus and PFC
Source: Front Pharmacol. 2020 Jan 17;10:1544. doi: 10.3389/fphar.2019.01544 (PMC6978986; doi:10.3389/fphar.2019.01544)

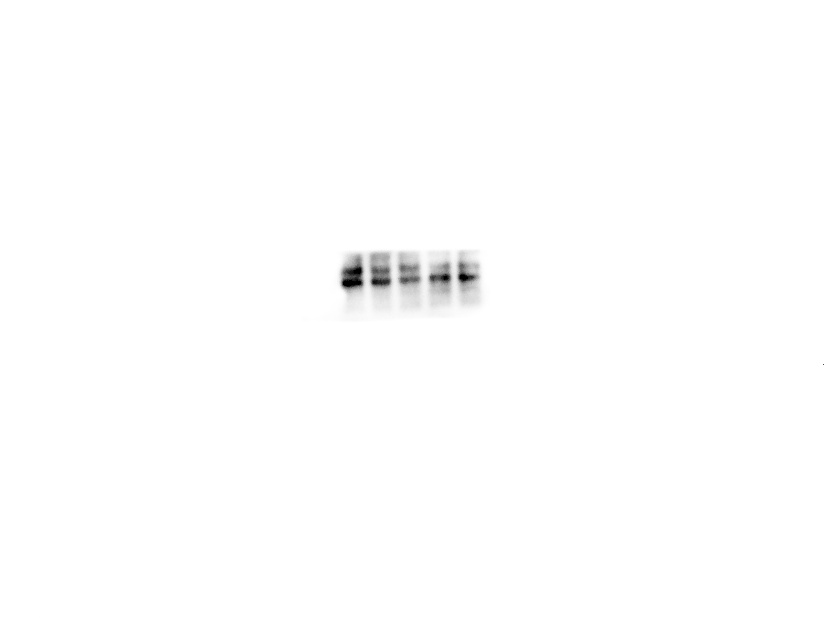

Supplement: Supplementary file 1 [file DataSheet_1.zip › supplementary materials ( original western blot figures)/BDNF(PFC).jpg]

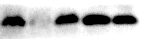

Supplement: Supplementary file 1 [file DataSheet_1.zip › supplementary materials ( original western blot figures)/BDNF(hippocampus).tif]

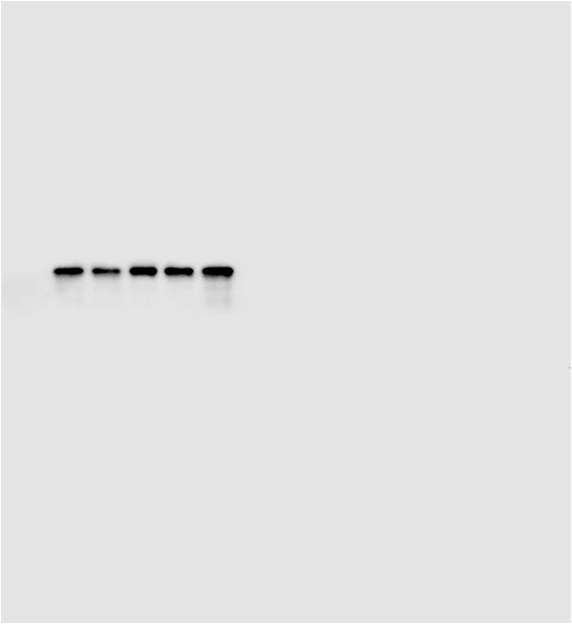

Supplement: Supplementary file 1 [file DataSheet_1.zip › supplementary materials ( original western blot figures)/Copine6 (PFC).tif]

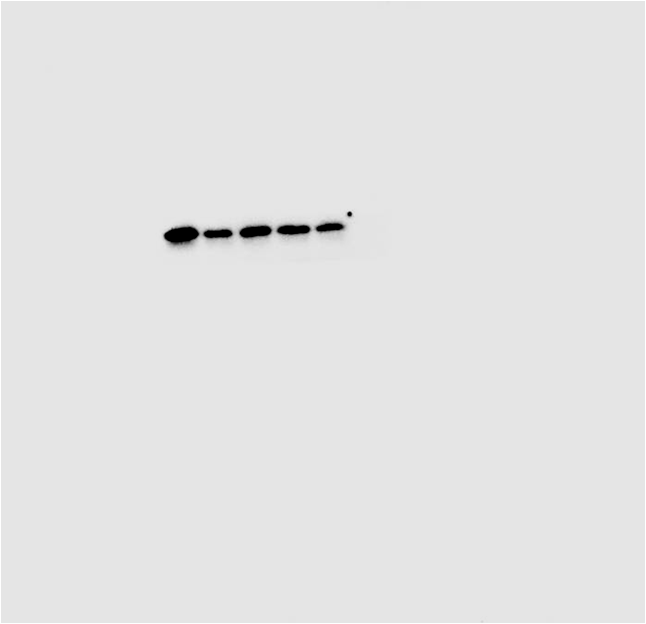

Supplement: Supplementary file 1 [file DataSheet_1.zip › supplementary materials ( original western blot figures)/Copine6 (hippocampus).tif]

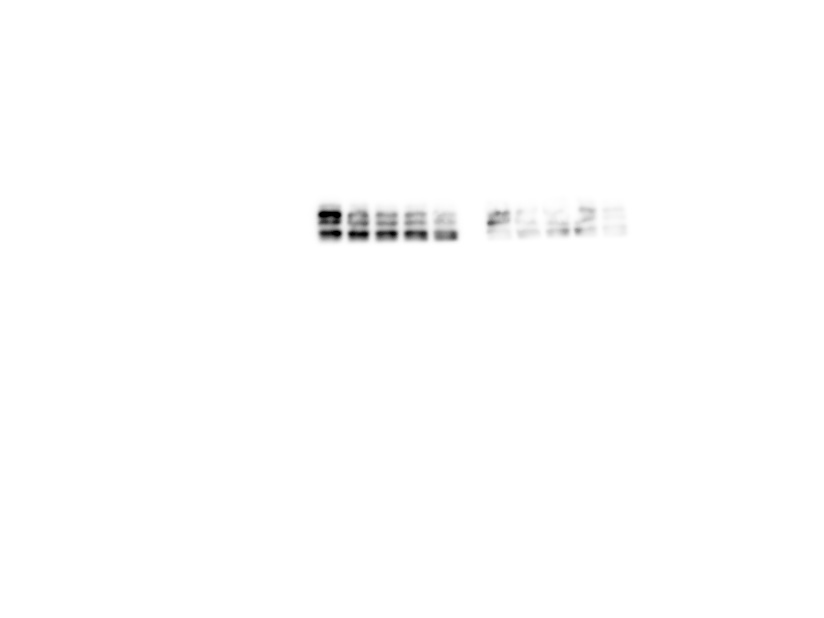

Supplement: Supplementary file 1 [file DataSheet_1.zip › supplementary materials ( original western blot figures)/Synapsin-1(PFC)-the left five samples are ours.jpg]

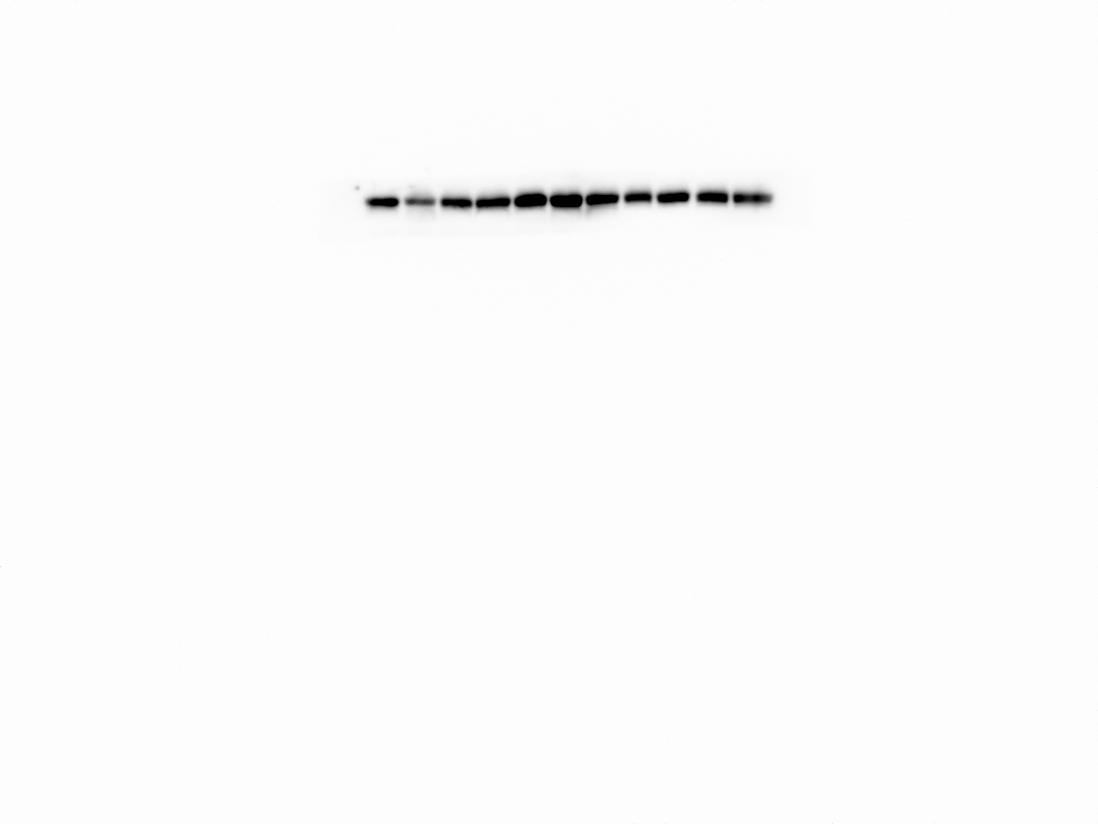

Supplement: Supplementary file 1 [file DataSheet_1.zip › supplementary materials ( original western blot figures)/Synapsin-1(hippocampus)-the right five samples are ours.tif]

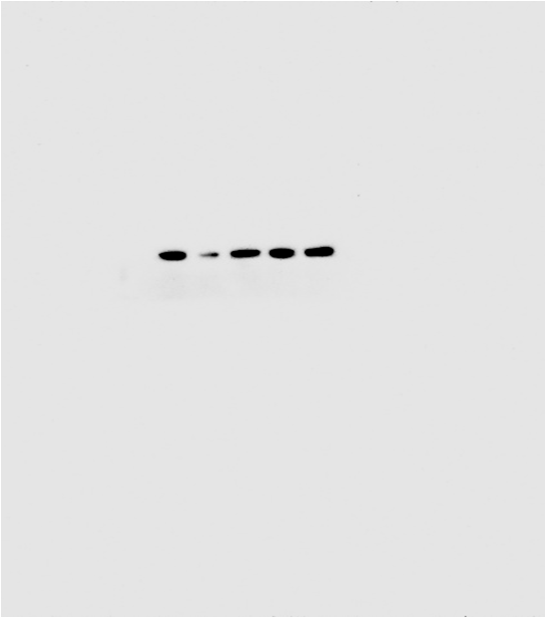

Supplement: Supplementary file 1 [file DataSheet_1.zip › supplementary materials ( original western blot figures)/TREM1 (PFC).tif]

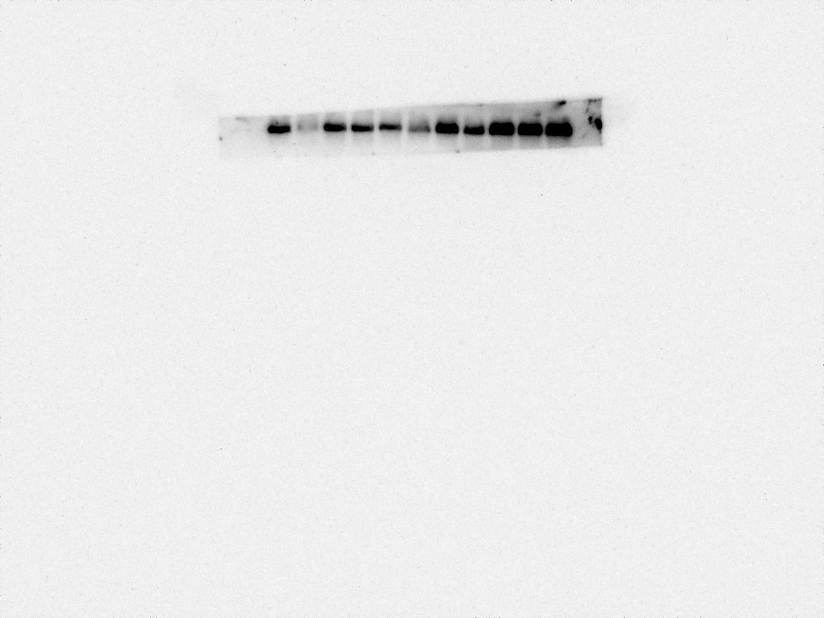

Supplement: Supplementary file 1 [file DataSheet_1.zip › supplementary materials ( original western blot figures)/TREM1 (hippocampus)-the right five samples are ours.tif]

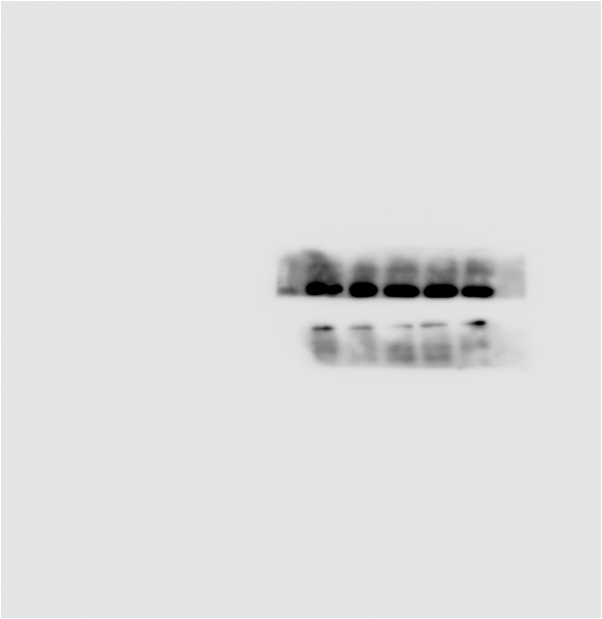

Supplement: Supplementary file 1 [file DataSheet_1.zip › supplementary materials ( original western blot figures)/TREM2 (hippocampus).tif]

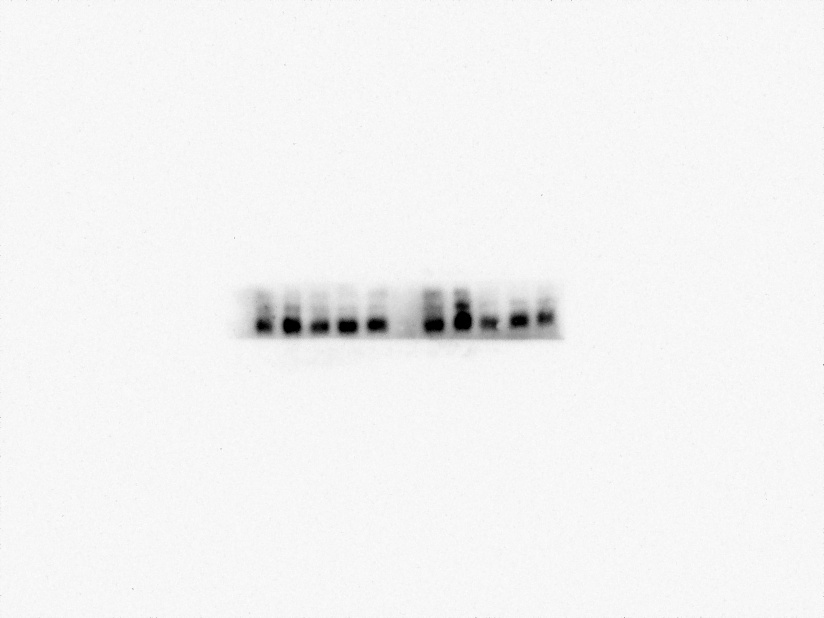

Supplement: Supplementary file 1 [file DataSheet_1.zip › supplementary materials ( original western blot figures)/TREM2(PFC)-the left five samples are ours.jpg]

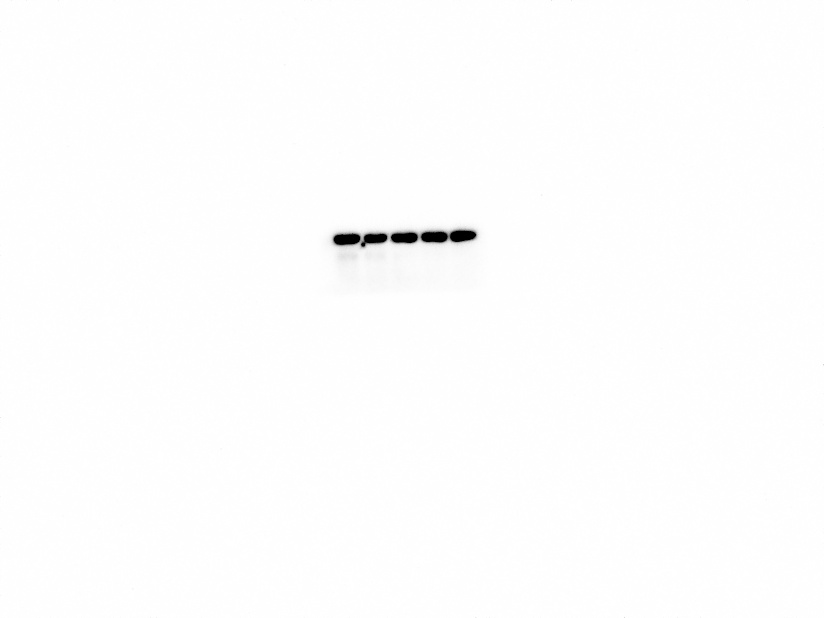

Supplement: Supplementary file 1 [file DataSheet_1.zip › supplementary materials ( original western blot figures)/actin (PFC).jpg]

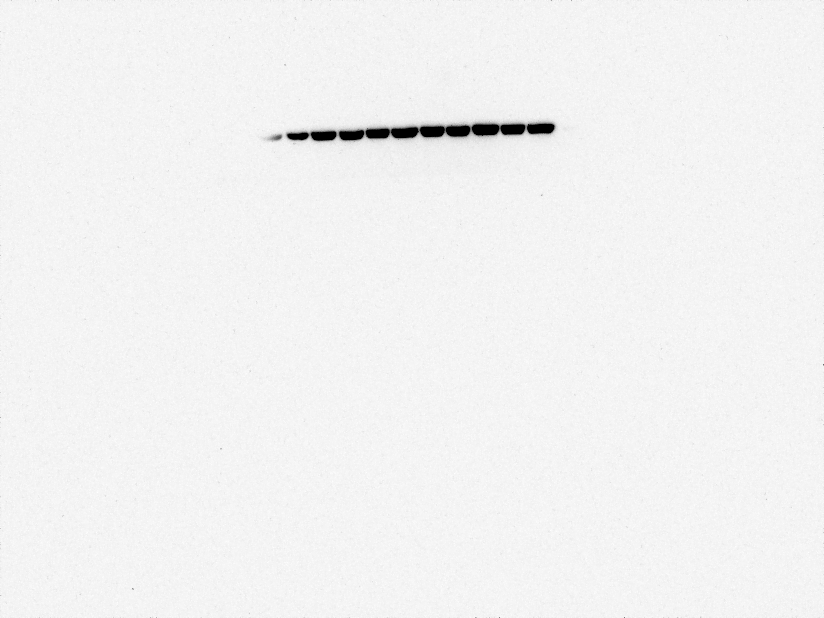

Supplement: Supplementary file 1 [file DataSheet_1.zip › supplementary materials ( original western blot figures)/actin (hippocampus)-the right five samples are ours.tif]

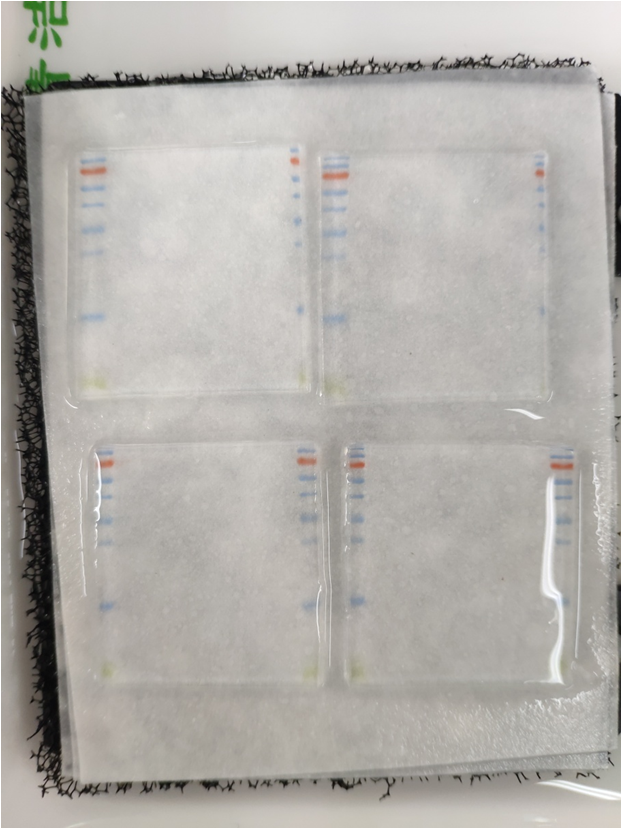

Supplement: Supplementary file 1 [file DataSheet_1.zip › supplementary materials ( original western blot figures)/gel with ladder (PFC).tif]

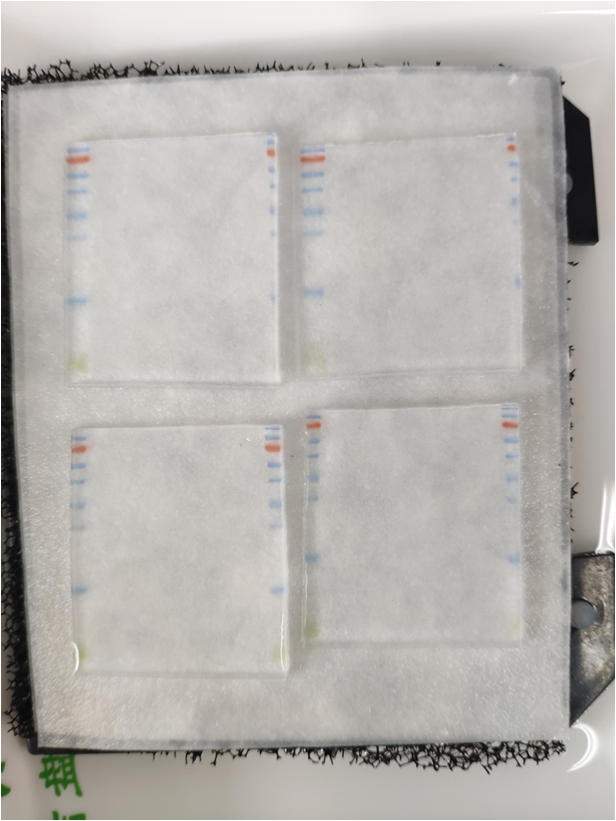

Supplement: Supplementary file 1 [file DataSheet_1.zip › supplementary materials ( original western blot figures)/gel with ladder (hippocapums).tif]
