# Supplementary material for: Quercetin Alleviates LPS-Induced Depression-Like Behavior in Rats via Regulating BDNF-Related Imbalance of Copine 6 and TREM1/2 in the Hippocampus and PFC
Source: Front Pharmacol. 2020 Jan 17;10:1544. doi: 10.3389/fphar.2019.01544 (PMC6978986; doi:10.3389/fphar.2019.01544)
Supplement: Supplementary file 2 [file Image_1.pdf]

Supplementary materials:

Legends of the figure

Fig. S1 effect of quercetin on the performance of mice in the forced swimming test and tail suspending test

The data are presented as the mean ± SEM, with 9 rats in each group.

Compared with that in the control group, the immobility time in the FST (A) and TST (B) was significant shorter in the quercetin-treated group.

$^{\#}P < 0.05$ and $^{\#\#}P < 0.001$ compared with the control group.

Fig. S1

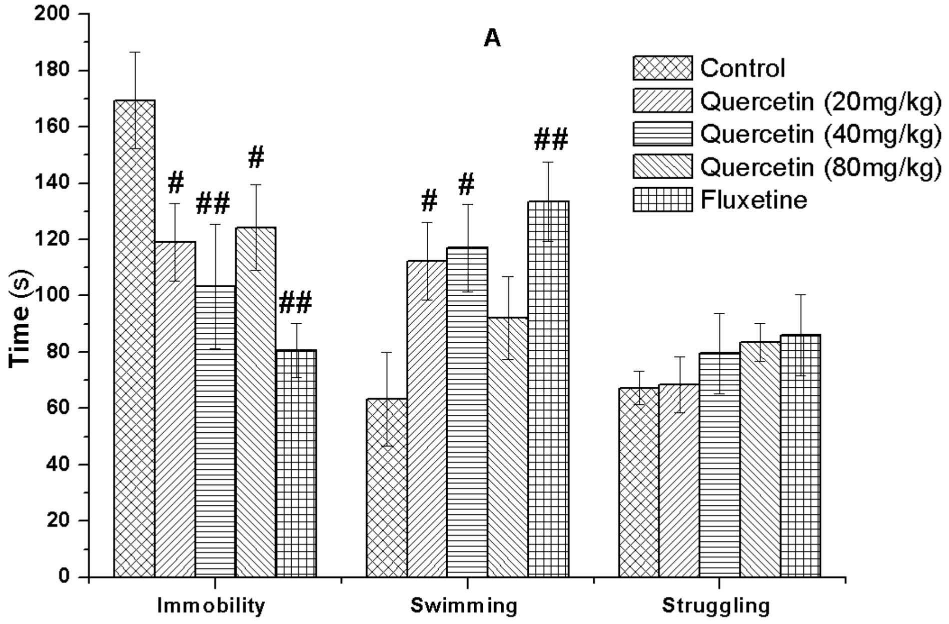

A
Control
Quercetin (20mg/kg)
Quercetin (40mg/kg)
Quercetin (80mg/kg)
Fluxetine
Time (s)
200
180
160
140
120
100
80
60
40
20
0
#
##
#
##
#
#
##
Immobility
Swimming
Struggling

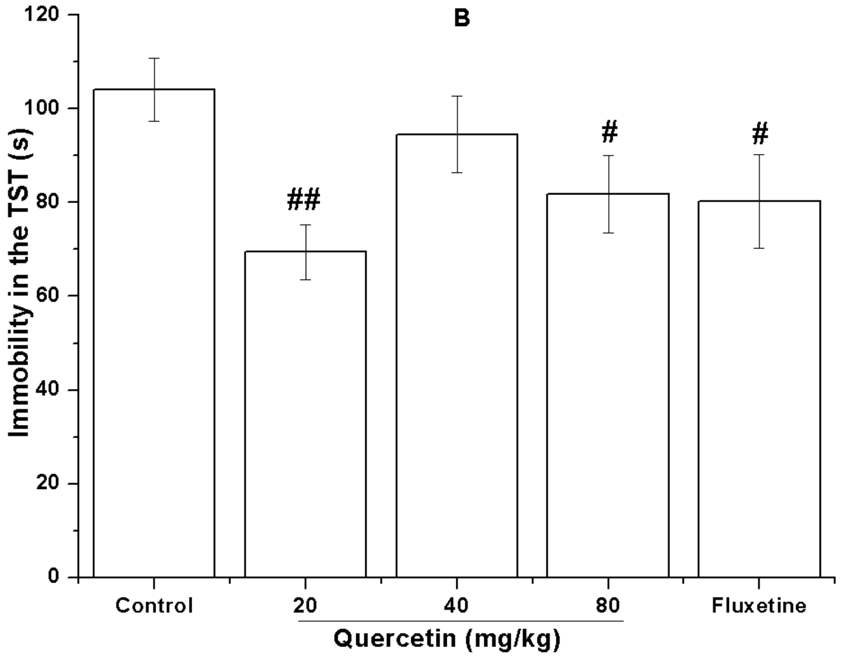

B
Immobility in the TST (s)
120
100
80
60
40
20
0
##
#
#
Control
20
40
80
Fluxetine
Quercetin (mg/kg)
